# The correlation between tinnitus-specific and quality of life questionnaires to assess the impact on the quality of life in tinnitus patients

**DOI:** 10.3389/fneur.2022.969978

**Published:** 2022-09-26

**Authors:** Lauren Van Hoof, Tobias Kleinjung, Emilie Cardon, Vincent Van Rompaey, Nicole Peter

**Affiliations:** ^1^Department of Otorhinolaryngology, Head and Neck Surgery, University Hospital Zurich, University of Zurich, Zurich, Switzerland; ^2^Department of Translational Neuroscience, Faculty of Medicine and Health Science, University of Antwerp, Edegem, Belgium; ^3^Department of Otorhinolaryngology and Head and Neck Surgery, Antwerp University Hospital, Edegem, Belgium

**Keywords:** tinnitus, quality of life, health related quality of life (HRQoL) questionnaires, Tinnitus Functional Index (TFI), Tinnitus Handicap Inventory (THI), world health organization quality-of-life (WHOQOL-BREF), 8-item short form health survey (SF-8)

## Abstract

**Introduction:**

Subjective tinnitus is often associated with a reduction in health-related quality of life (HRQoL). The HRQoL represents the impact of tinnitus on an individual's life by addressing the physical, social, and psychological domains of 1. A limited amount of studies has investigated the association between tinnitus and HRQoL questionnaires. The aim of this study was to examine the correlation between tinnitus-specific and HRQoL questionnaires in order to shorten fulfilling questionnaires, as it is often time-consuming.

**Material and method:**

Eighty-five patients with tinnitus as primary complaint completed five questionnaires, including one general, two tinnitus-specific, and two generic HRQoL questionnaires: Tinnitus Sample Case History Questionnaire (TSCHQ), Tinnitus Functional Index (TFI), Tinnitus Handicap Inventory (THI), short version of World Health Organization Quality of Life (WHOQOL-BREF), and the eight-item Short-Form (SF-8). Four simple linear regression models were used to analyze the relationship between the THI and TFI and the WHOQOL-BREF and SF-8.

**Results:**

A negative and strong correlation was found between the tinnitus questionnaires and the SF-8. More than half of the variability in the SF-8 scores could be explained by the TFI and THI, respectively 50.6 and 54.4% (all *p* < 0.001). A strong negative regression was also found between the WHOQOL-BREF and the THI and TFI with a decrease in the determination coefficient of approximately 10% compared with the SF-8. The weakest correlation (regression coefficient of 0.628, *p* < 0.001) was observed between the WHOQOL-BREF and the TFI, indicating that the WHOQOL-BREF mean score explained 39.4% of the TFI. When looking at the subdomain scores, a strong correlation was observed between the QoL subdomain of the TFI and a combination of the physical and psychological subdomain of the WHOQOL-BREF (*r* = −0.627, *p* < 0.001).

**Conclusion:**

The QoL subdomain of the TFI gives good information about the physical and psychological health. Thus, the TFI is suitable to assess both tinnitus severity and the HRQoL. The coefficients of determination of the WHOQOL-BREF were significantly lower compared to the SF-8, suggesting that the WHOQOL-BREF provides more specific information about HRQoL. If more specific information on HRQoL, such as “environment” and “social relationships”, is required, it is recommended to use the WHOQOL-BREF.

## Introduction

Tinnitus is the perception of sound without the presence of an external sound source. It is a highly prevalent disorder, and ~10–15% of the adult population suffers from chronic, subjective tinnitus (1–4). The heterogeneous disorder is experienced differently in every individual: some only experience little discomfort, while others feel a great impact on cognitive abilities and emotional aspects (5, 6). The latter is associated with a reduced health-related quality of life (HRQoL) in tinnitus sufferers. The concept of HRQoL focuses on the impact that a certain health status has on an individual's life by looking at the physical, social, and psychological aspects of health (7). Decreased HRQoL is often caused by additional complaints beyond the tinnitus sound such as elevated stress levels, hearing difficulties, concentration problems, and sleep disturbances (8). Furthermore, there is a high comorbidity between chronic tinnitus and psychiatric disorders, such as anxiety and depression (9). Concurrent psychiatric disorders affect the severity or tolerance of tinnitus, resulting in a decrease in HRQoL in tinnitus patients.

The HRQoL is represented by utility scores that refer to the preferred health state of patients (10). Measuring HRQoL is useful for assessing the burden of tinnitus, detecting hidden or unexpected health problems, and identifying health inequalities among patient groups. In this study, indirect methods including the abbreviated version of the World Health Organization Quality of Life Survey (*WHOQOL-BREF*) (11) and the eight-item short form (*SF-8*) (12) were used as HRQoL questionnaires. Two tinnitus-specific questionnaires were used to assess the tinnitus severity, namely the Tinnitus Functional Index (TFI) (13) and Tinnitus Handicap Inventory (THI) (14). In this study, the German versions of these questionnaires were applied, which are all standardized and validated (11–15). The HRQoL in tinnitus patients is a widely impacting and important topic but there is still a limited amount of knowledge about the effect of tinnitus on the HRQoL. According to a search of the literature, few studies were found that considered both tinnitus-specific questionnaires and HRQoL questionnaires. Thus, there is insufficient information on the relationship between tinnitus questionnaires and HRQoL questionnaires to determine the added value of HRQoL questionnaires.

The aim of this study was to investigate whether there is an efficient way to evaluate the impact of tinnitus on the HRQoL by comparing the tinnitus-specific questionnaires (*TFI* and *THI*) with the HRQoL questionnaires (*WHOQOL-BREF* and *SF-8*). More specifically, the main objective was to investigate the correlation between the tinnitus-specific questionnaires (*TFI* and *THI*) and the HRQoL questionnaires (*WHOQOL-BREF* and *SF-8*) to investigate if the HRQoL questionnaires provide additional benefit or if the HRQoL is already well assessed with the tinnitus questionnaires. The hypothesis is that there is a strong correlation between the tinnitus questionnaires and the HRQoL questionnaires. In contrast to the THI, the TFI has a subdomain that should represent QoL. As secondary objective, the subdomain QoL of the TFI was analyzed to determine which aspects of the HRQoL were included in this subdomain by comparing the QoL subdomain of the TFI to the four broad domains of the *WHOQOL-BREF* (physical health, psychological health, social relationships, and environment). The observed information may impact the evaluation of HRQoL for future tinnitus research and clinical practice.

## Materials and methods

### Study setting and patients

In this study, questionnaires completed by patients in the tinnitus clinic at the Department of Otorhinolaryngology—Head and Neck Surgery at the University Hospital of Zurich (USZ) were analyzed prospectively. The study was approved by the Ethical Committee of the Canton of Zürich (BASEC-Nr: 2021-00361). Only adult patients (male and female) diagnosed with tinnitus as a primary complaint were included. Furthermore, patients had to have sufficient knowledge of the German language and computer skills to complete the self-report questionnaires (one general questionnaire, two questionnaires measuring the tinnitus burden, and two assessing the HRQoL in patients). The assessment of the tinnitus-related handicap was executed by the *TFI* (16) and *THI* (17). To measure the impact of tinnitus on the HRQoL in tinnitus patients, the *WHOQOL-BREF* (11) and *SF-8* (18) questionnaires were used. All questionnaires were sent to the patients via an online tool (Innoforce ENT Statistics, www.innoforce.com) a few days before the first consultation in the tinnitus clinic.

### Assessment

#### Tinnitus Sample Case History Questionnaire

One general questionnaire, the *Tinnitus Sample Case History Questionnaire (TSCHQ)*, was used to collect data of the patient, including tinnitus history, previous treatments, general hearing problems, impact on HRQoL, and general health status by answering a total of 35 items (19). The validated German version of the TSCHQ was used in this study to obtain background information about the patient's tinnitus (20).

#### Tinnitus Handicap Inventory

The *Tinnitus Handicap Inventory* [*THI*, (17)] is a widely used instrument that investigates three different domains: functional limitations, emotional response, and catastrophic aspects, containing, respectively eleven, nine, and five items. Patients are asked to complete the validated German version of the questionnaire containing 25 statements. Each statement can be answered by yes (four points), sometimes (two points), and no (zero points) (21). The total sum score of the questionnaire indicates the severity of the complaints, with a score of 100 representing the greatest suffering from tinnitus. Five levels of total scores can be differentiated: low handicap (0–16), mild handicap (18–36), moderate handicap (38–56), severe handicap (58–76), and catastrophic handicap (78–100) (17).

#### Tinnitus Functional Index

To scale the overall tinnitus severity, we used the *Tinnitus Functional Index* [*TFI*, (16)], which promises to be the new gold standard for tinnitus assessment (22). In addition, this more recent questionnaire was especially designed to evaluate different tinnitus treatments. In this study, we used the validated German version of the *TFI* (13, 15). The questionnaire consists of 25 items divided into eight subscales: intrusiveness, reduced sense of control, cognitive interference, sleep disturbance, auditory difficulties, interference with relaxation, quality of life, and emotional distress. Each subscale consists of three questions, with the exception of the subdomain “Quality of Life”, which has four items. By answering each question on an 11-point Likert scale ranging from zero to ten, patients assess how they felt over the past week. Exceptions are questions 1 and 3, as these represent a response scale of 0 to 100% in steps of ten percent, which were transformed into a 0 to 10 scale for further calculations. The total score ranges from zero to 100, with a higher score indicating a greater impact of tinnitus on the patient's daily life. The tinnitus severity can be divided into five categories: not a problem (mean = 14, range 0–17), small problem (mean = 21, range 18–31), moderate problem (mean = 42, range 32–53), big problem (mean = 65, range 54–72) or very big problem (mean = 78, range 73–100) (2).

#### WHOQOL-BREF

In order to assess the HRQoL, tinnitus sufferers are asked to complete the abbreviated German version of the *World Health Organization Quality of Life Survey* [*WHOQOL-BREF*, (11)]. This questionnaire contains 26 items, each rated on a 5-point Likert scale, of which two items are examined separately. The two individual items represent the patient's overall perception of their HRQoL and the overall perception of their general health. The other 24 questions are divided into four broad domains, including physical health, psychological health, social relationships, and environment. A higher total domain score corresponds to a better HRQoL within that domain (11).

#### Eight-item Short-Form

The *eight-item Short-Form* (*SF-8*) is an abbreviated version of the SF-36, a widely-used questionnaire measuring the general health status (12, 18). The *SF-8*, consisting of eight items, validates the HRQoL profile with regard to physical, psychological, and social aspects. Each item represents one of the eight SF-36 domains: physical functioning (PF), role limitation due to physical problems (RP), body pain (BP), general health (GH), vitality (VT), social functioning (SF), role limitation due to emotional problems (RE), and mental health (MH). For each of the eight questions, patients are asked to answer on a 5-point Likert scale how much the tinnitus affects their daily lives. The *SF-8* is assessed by two summary scores consisting of a physical component (PF, RP, BP, and GH) and a mental health component (VT, SF, RE, and MH). A higher *SF-8* score represents less disability with eight being the maximum disability and 40 the minimum disability (23).

### Statistical analysis

A linear regression model using the least square method was used to analyze the effect of the tinnitus questionnaires on the HRQoL questionnaires. First, general characteristics of the study participants collected by the TSCHQ were summarized as mean values and standard deviations (SD). Normal distributions for the dependent and independent variables were observed using the Shapiro-Wilk's test (*p* > 0.0125) (24) and a visual inspection of their histograms, normal Q-Q plots and box plots. Then, multiple testing was performed with the *THI* and *TFI* as independent predictors and the *WHOQOL-BREF* and *SF-8* as response outcomes. Here, four separate simple linear regressions were calculated: the effect of *TFI* on *WHOQOL-BREF* and on *SF-8*, the effect of *THI* on *WHOQOL-BREF*, and on *SF-8*. After adjustment for multiple testing, a *p*-value < 0.0125 was considered statistically significant. Residual normality, homoscedasticity and removal of outliers was checked using visual inspection of their histograms, normal Q-Q plots and scatterplots. An analysis of the data was performed using the statistical program SPSS (Statistical Package for Social Sciences, version 26.0, IBM, USA). The Tukey honestly significant difference (HSD) test was used to calculate the difference in HRQoL between all grades of tinnitus severity. This frequently used pairwise comparison technique calculates the HSD between two means using a statistical distribution that gives the exact sampling distribution of the largest difference between a set of means originating from the same population (25).

## Results

### Demographic characteristics

From the 134 individuals that visited the clinic, 85 participants met all criteria and were included in the analysis. Forty-nine patients could not be included because they did not have sufficient knowledge of the German language (*n* = 15), not enough computer skills (*n* = 3), refused to participate in the study (*n* = 12), informed consent was not obtained (*n* = 5), or did not complete all five questionnaires (*n* = 14). The mean age of the participants was 51.6 years (±14.3 SD, range 21–85 years). Fifty-one (60.0%) participants were male and 34 (40.0%) individuals were female. Of the 85 participants, 51 (60.0%) experienced tinnitus on both sides, 17 (20.0%) individuals only on the left, and 17 (20.0%) only on the right side. Additionally, the mean duration of tinnitus of the study population was 7.0 years (±8.4 SD, range 0–37 years) and 61.2% patients had been experiencing tinnitus between 0 and 4 years. Overall, 69.4% of the study population had a normal hearing and only 3.5% suffered from severe hearing loss. Further demographic and clinical characteristics can be found in Table 1.

**Table 1:** Demographic and tinnitus characteristics of all participants.

		**Mean (SD)**	**Count (%)**
**Age (years)**		51.6 (14.3)	
**Gender**			
	Male		51 (60.0)
	Female		34 (40.0)
**Tinnitus side**			
	Bilateral		51 (60.0)
	Left		17 (20.0)
	Right		17 (20.0)
**Duration (years)**		7.0 (8.4)	
**Frequency of tinnitus sound**			
	Low		11 (12.9)
	Moderate		15 (17.7)
	High		32 (37.7)
	Very high		27 (31.8)
**Level of hearing loss (PTA 500–4,000 k Hz)**		20.2 (16.5)	
	Normal hearing		59 (69.4)
	Mild hearing loss		13 (15.3)
	Moderate hearing loss		10 (11.8)
	Severe hearing loss		3 (3.5)

**Figure 1:**
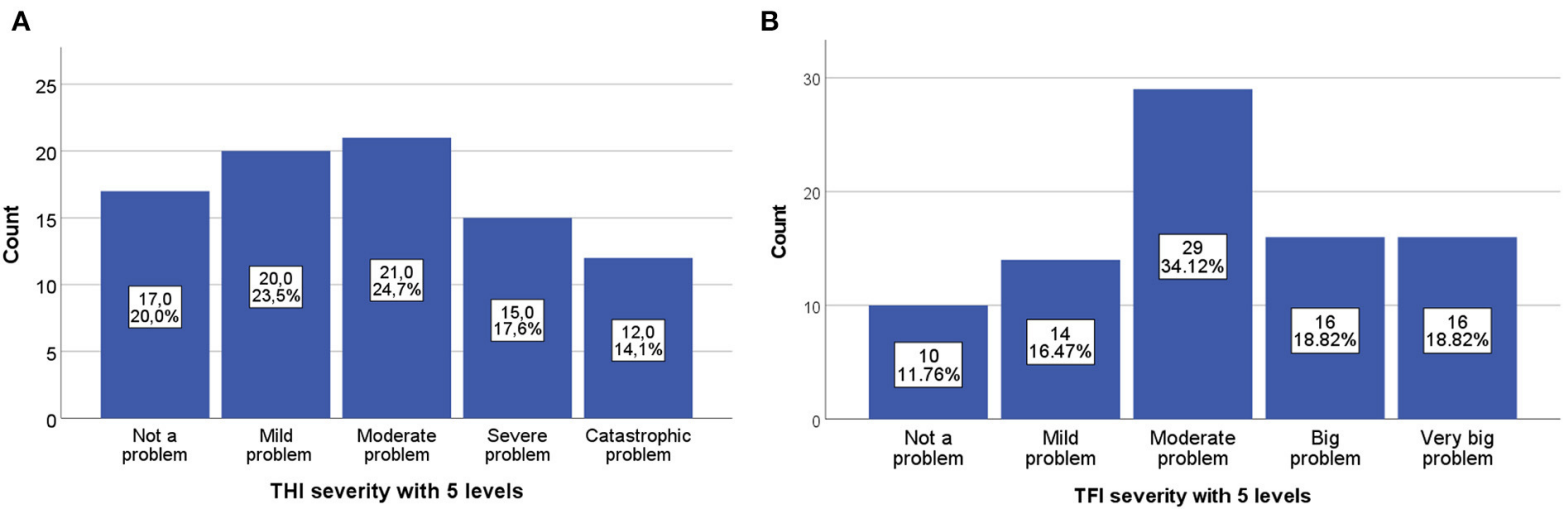
Distribution of the tinnitus severity of the tinnitus questionnaires. **(A)** Tinnitus severity measured by the THI divided in 5 levels. **(B)** Tinnitus severity measured by the TFI divided in 5 levels.

The distribution of the tinnitus severity according to the tinnitus questionnaires, divided into five categories is shown in Figure 1. For the *THI* most patients were categorized in the group with moderate problems, followed by mild and slight problems (Figure 1A). For the *TFI*, the largest category was also the group with moderate problems (Figure 1B).

### Overall and subdomain scores of the different questionnaires

All mean scores and SDs of the different questionnaires and their subdomains are demonstrated in Table 2. The mean total scores of the *THI* and *TFI* were 43.3 (SD = 25.7) and 48.0 (SD = 24.1) respectively, which represents a moderate tinnitus severity with a broad range. The distribution of the total scores of both the *TFI* and *THI* are shown in Figure 1. The results of both tinnitus questionnaires and both QoL questionnaires were normally distributed. Also, checking for normality of the residuals was normal, homoscedasticity of the results was observed, and there were no outliers. The mean total score of *WHOQOL-BREF* was 68.1 (SD = 15.3). The mean scores of the subdomains general health, physical, psychological, social relationships, and environment, were all above 50%, indicating an overall good HRQoL in our sample. The mean *SF-8* total score was 29.1 (SD = 7.3), which indicated a good HRQoL in our tinnitus population. This can also be confirmed by the physical and mental component that had mean scores of 14.4 (SD = 4.2) and 14.7 (SD = 3.9), respectively. These scores suggested a good physical and mental health.

**Table 2:** Distribution of mean and SD scores of all questionnaires and their subdomains.

	**Mean**	**SD**
**Tinnitus handicap inventory (** ***THI*** **)**		
Total score (0–100)	**43.3**	**25.7**
Functional limitations (0–44)	18.7	12.0
Emotional response (0–36)	14.5	10.0
Catastrophic aspects (0–20)	10.1	5.5
**Tinnitus functional index (** ***TFI*** **)**		
Total score (0–100)	**48.0**	**24.1**
Intrusiveness (0–100)	59.5	24.4
Sense of control (0–100)	58.5	25.6
Cognitive complaints (0–100)	43.7	27.4
Sleep disturbance (0–100)	47.6	33.5
Auditory difficulties (0–100)	36.4	31.3
Relaxation (0–100)	53.5	29.8
Quality of Life (0–100)	39.4	28.0
Emotional distress (0–100)	47.9	28.2
**World Health organization QoL (** ***WHOQOL-BREF*** **)**		
Mean score (0–100)	**68.1**	**15.5**
General health (0–100)	57.4	24.0
Physical (0–100)	66.6	19.8
Psychological (0–100)	65.5	18.7
Social relationships (0–100)	71.4	18.7
Environment (0–100)	79.6	13.6
**Short form 8 (** ***SF-8*** **)**		
Total score (0–40)	**29.1**	**7.3**
Physical component (0–20)	14.4	4.2
Mental component (0–20)	14.7	3.9

All total values are presented in bold.

### Correlations between both tinnitus questionnaires and HRQoL questionnaires (THI vs TFI and WHOQOL-BREF vs SF-8)

First, the correlations between both groups of questionnaires were calculated (*THI* vs. *TFI, WHOQOL-BREF* vs. *SF-8*). The correlation between the two tinnitus questionnaires *THI* and *TFI* was positive and very strong (*r* = 0.864, *p* < 0.001, *N* = 85). For the HRQoL questionnaires, multiple correlations were calculated, because the *WHOQOL-BREF* has no overall score, only subdomain scores. To compare the HRQoL scores of the *WHOQOL-BREF* with the *SF-8* questionnaire, one overall *WHOQOL-BREF* score was necessary. Therefore, an overall *WHOQOL-BREF* score was calculated where the mean of all subdomains was used:

$$\begin{array}{l} \frac{\begin{array}{c} mean\;of\;general\;health\;+\;mean\;of\;physical\;subdomain\\ +\;mean\;of\;psychological\;subdomain\;+\;mean\;of\;social\;relationships\\ +\;mean\;of\;environment \end{array}}{5}\\ =overall\;WHOQOL-BREF. \end{array}$$

**Figure 2:**
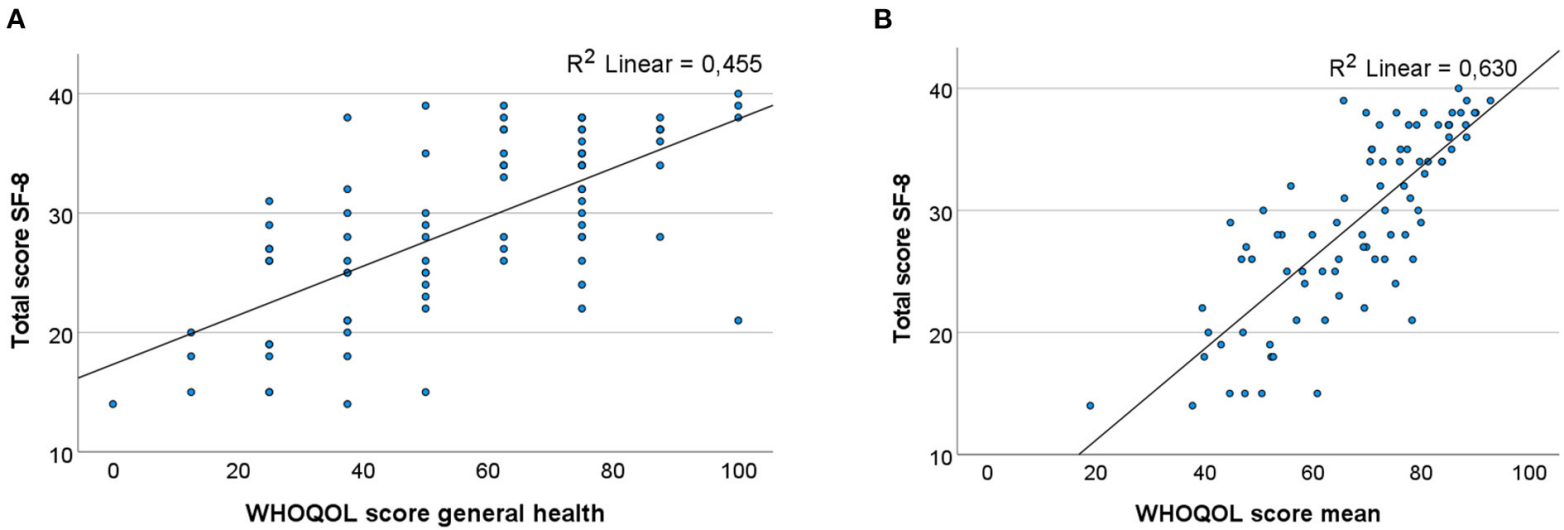
**(A)** Scatterplot of the correlation between WHOQOL-BREF general health and the total score of the SF-8 (*r* = 0.675, *p* < 0.001). **(B)** Scatterplot of the correlation between the WHOQOL-BREF mean score and the SF-8 total score (*r* = 0.794, *p* < 0.001).

A strong correlation was observed between the *WHOQOL-BREF* mean score and the total *SF-8* score (*r* = 0.794, *p* < 0.001, *N* = 85). In addition, the correlation of the subdomain general health (GH) of the *WHOQOL-BREF*, consisting of two questions, with the *SF-8* had a positive and moderate correlation (*r* = 0.675, *p* < 0.001, *N* = 85). Linear relationships are presented in a scatterplot as shown in Figure 2. These scatterplots confirm the strong correlation between the *WHOQOL-BREF* mean score and the *SF-8*. Since the GH subdomain only consists of two questions and has a weaker correlation, the *WHOQOL-BREF* mean score was used for further calculations as overall HRQoL score.

### Linear regressions between the tinnitus questionnaires and the HRQoL questionnaires: Primary outcomes

**Figure 3:**
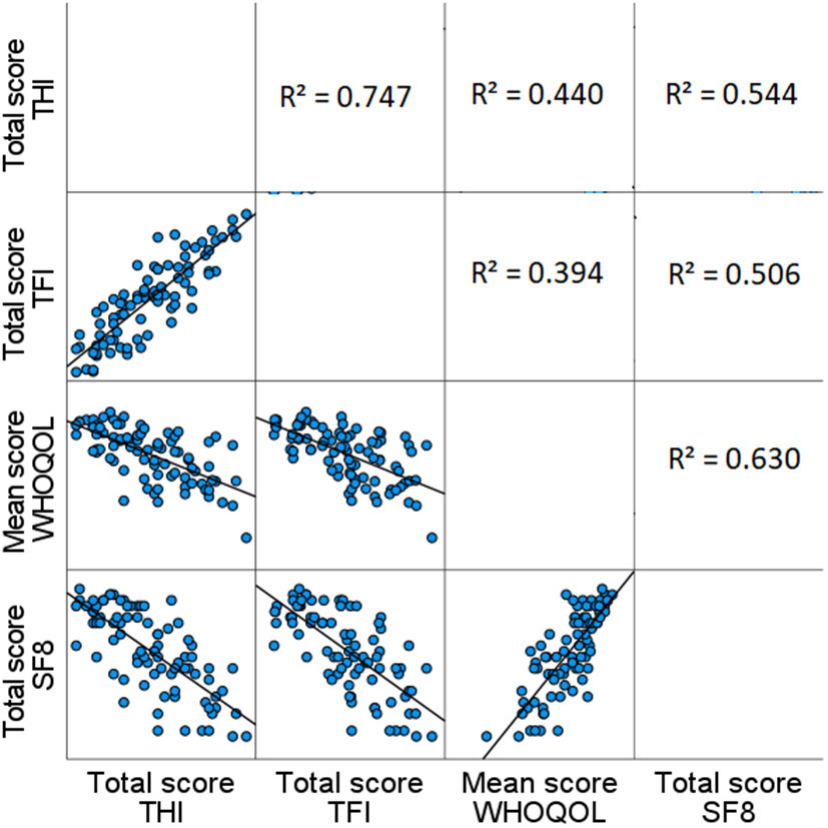
Scatterplots and determination coefficients of TFI, THI, WHOQOL-BREF, and SF-8. The scatterplots and determination coefficients (*R*^2^) show the strength of the regressions between the TFI, THI, WHOQOL-BREF, and SF-8. In the left corner below, the scatterplots are presented, whereas the determination coefficients are shown in the top right corner.

The primary objective of this study was to investigate if the HRQoL questionnaires have an additional benefit as compared to the tinnitus questionnaires. After calculating four simple linear regressions, all combinations of questionnaires were significant. The linear regression with the *SF-8* as dependent variable and the *TFI* as independent outcome showed a strong correlation with a regression coefficient of 0.711 (b = −0.711 [98.75% confidence interval], CI −0.908 and −0.514, *R*^2^ = 0.506, *p* < 0.001, F = 85.000). The strongest regression was between the THI and SF-8 with a regression coefficient of 0.738 (b = −0.738 [98.75% confidence interval], CI −0.927 and −0.548, *R*^2^ = 0.544, *p* < 0.001, F = 99.101). In addition, the determination coefficient (*R*^2^) of both the TFI and THI correlated with the SF-8 was strong and exceeded 50% (Figure 3). This indicates that more than half of the variability in the *SF-8* scores can be explained by the model's input. When the *WHOQOL-BREF* mean was used as dependent variable, a moderate correlation was observed (Figure 3). The weakest correlation occurred between the *WHOQOL-BREF* and the *TFI*, which had a moderate regression coefficient of 0.628 (b = −0.628 [98.75% confidence interval], CI −0.846 and −0.410, R^2^ = 0.394, *p* < 0.001, F = 53.996). Similarly, a moderate regression coefficient of 0.664 was observed between the *WHOQOL-BREF* and the *THI* (b = −0.664 [98.75% confidence interval], CI −0.873 and −0.454, *R*^2^ = 0.440, *p* < 0.001, F = 65.312). Here, the determination coefficient showed that the score of the *WHOQOL-BREF* mean was explained for 39.4 and 44.0% by the *TFI* and *THI* respectively, which was slightly decreased compared to the *SF-8* questionnaire with the tinnitus questionnaires. When observing the difference between the *TFI* and *THI*, a slightly higher regression coefficient is noticed when the *THI* is used as an independent outcome. After calculating multiple regressions that included putative confounding factors such as the duration of tinnitus, age and PTA scores, no significant contribution of these factors was observed. Thus, the final model did not include any of these factors.

### Linear regressions between the tinnitus questionnaires and subdomains of the HRQoL questionnaires

Furthermore, the relationships between the tinnitus questionnaires and all the subdomains of the HRQoL questionnaires were calculated (Table 3). Both the *TFI* and the *THI* related to the mental component of the *SF-8* had a strong regression coefficient, respectively 0.715 (R^2^ = 0.511, *p* < 0.001, F = 86.842) and 0.793 (*R*^2^ = 0.629, *p* < 0.001, F = 140.913). A weak relationship was observed between the *TFI* and the social relationships subdomain of the *WHOQOL-BREF* with a regression coefficient of 0.217 (*R*^2^ = 0.047, *p* = 0.046, F = 4.116). In addition, the regression between the social relationships subdomain and the *THI* was weak with a regression coefficient of 0.246 (*R*^2^ = 0.060, *p* = 0.023, F = 5.332). If the *TFI* values were compared to the *THI* values, no big differences were observed.

**Table 3:** Linear regressions between the tinnitus questionnaires and all subdomains of the HRQoL questionnaires.

	***p*-value**	***R***	***R* ^2^**	***F*-value**
*TFI* vs *SF-8* physical	<0.001	0.586	0.343	43.347
*TFI* vs *SF-8* mental	<0.001	0.715	0.511	86.842
*TFI* vs *WHOQOL-BREF* physical	<0.001	0.635	0.403	55.992
*TFI* vs *WHOQOL-BREF* psychological	<0.001	0.628	0.395	54.094
*TFI* vs *WHOQOL-BREF* social relationships	0.046	0.217	0.047	4.116
*TFI* vs *WHOQOL-BREF* environment	<0.001	0.483	0.234	25.315
*THI* vs. *SF-8* physical	<0.001	0.559	0.313	37.821
*THI* vs. *SF-8* mental	<0.001	0.793	0.629	140.813
*THI* vs. *WHOQOL-BREF* physical	<0.001	0.629	0.395	54.242
*THI* vs. *WHOQOL-BREF* psychological	<0.001	0.679	0.461	70.854
*THI* vs. *WHOQOL-BREF* social relationships	0.023	0.246	0.060	5.332
*THI* vs. *WHOQOL-BREF* environment	<0.001	0.584	0.341	43.004

### The correlations between the QoL subdomain of the TFI and different subdomains of HRQoL questionnaires

As described previously, the *TFI* exists of different subdomains, which also include a QoL sub-score. We studied what kind of information this subdomain evaluates in terms of HRQoL assessment. The correlation coefficients are shown in Table 4. Since a higher score of the *TFI* QoL represents a worse HRQoL, a negative correlation was expected. Only between the *TFI* QoL and the subdomain social relationships of the *WHOQOL-BREF* a very weak and non-significant correlation was observed (r = −0.183, *p* = 0.094). All other correlations were significant. Between the *TFI* QoL and a combination of the physical and psychological subdomain of the *WHOQOL-BREF*, a strong correlation was observed (*r* = −0.627, *p* < 0.001). There was also a strong correlation between the *TFI* QoL subdomain and the total score of the *SF-8* (*r* = −0.703, *p* < 0.001).

**Table 4:** Correlations between QoL subdomain of *TFI* and all subdomains of HRQoL questionnaires (*WHOQOL-BREF* and *SF-8*).

	***p*-value**	**Correlation coefficient**
*TFI* QoL vs. *WHOQOL-BREF* mean	<0.001	−0.584
*TFI* QoL vs. *WHOQOL-BREF* physical	<0.001	−0.605
*TFI* QoL vs. *WHOQOL-BREF* psychological	<0.001	−0.570
*TFI* QoL vs. *WHOQOL-BREF* social relationships	0.094	−0.183
*TFI* QoL vs. *WHOQOL-BREF* environment	<0.001	−0.453
*TFI* QoL vs. *WHOQOL-BREF* general health	<0.001	−0.547
*TFI* QoL vs. *WHOQOL-BREF* phy + psy	<0.001	–**0.627**
*TFI* QoL vs. *WHOQOL-BREF* phy + psy + env	<0.001	−0.618
*TFI* QoL vs. *WHOQOL-BREF* phy + psy + GH	<0.001	−0.630
*TFI* QoL vs. *WHOQOL-BREF* phy + psy + env + GH	<0.001	−0.630
*TFI* QoL vs. *SF-8*	<0.001	–**0.703**
*TFI* QoL vs. *SF-8* physical component	<0.001	−0.573
*TFI* QoL vs. *SF-8* mental component	<0.001	−0.713

Phy, physical; psy, psychological; env, environment; GH, general health. The correlations of interest are shown in bold.

### The mean scores of the HRQoL questionnaires in proportion with the tinnitus severity

We also compared the HRQoL scores by looking at the grade of tinnitus severity. As the tinnitus severity increased (a higher *TFI* or *THI* grade), the HRQoL score decreased. The score of the *WHOQOL-BREF* mean is presented on a scale of 100 compared to a scale of 40 for the *SF-8*. A noticeable decrease in HRQoL was observed when tinnitus becomes a moderate problem or higher. When tinnitus severity was in the highest level (very big problem), the decline in HRQoL was even more pronounced.

**Figure 4:**
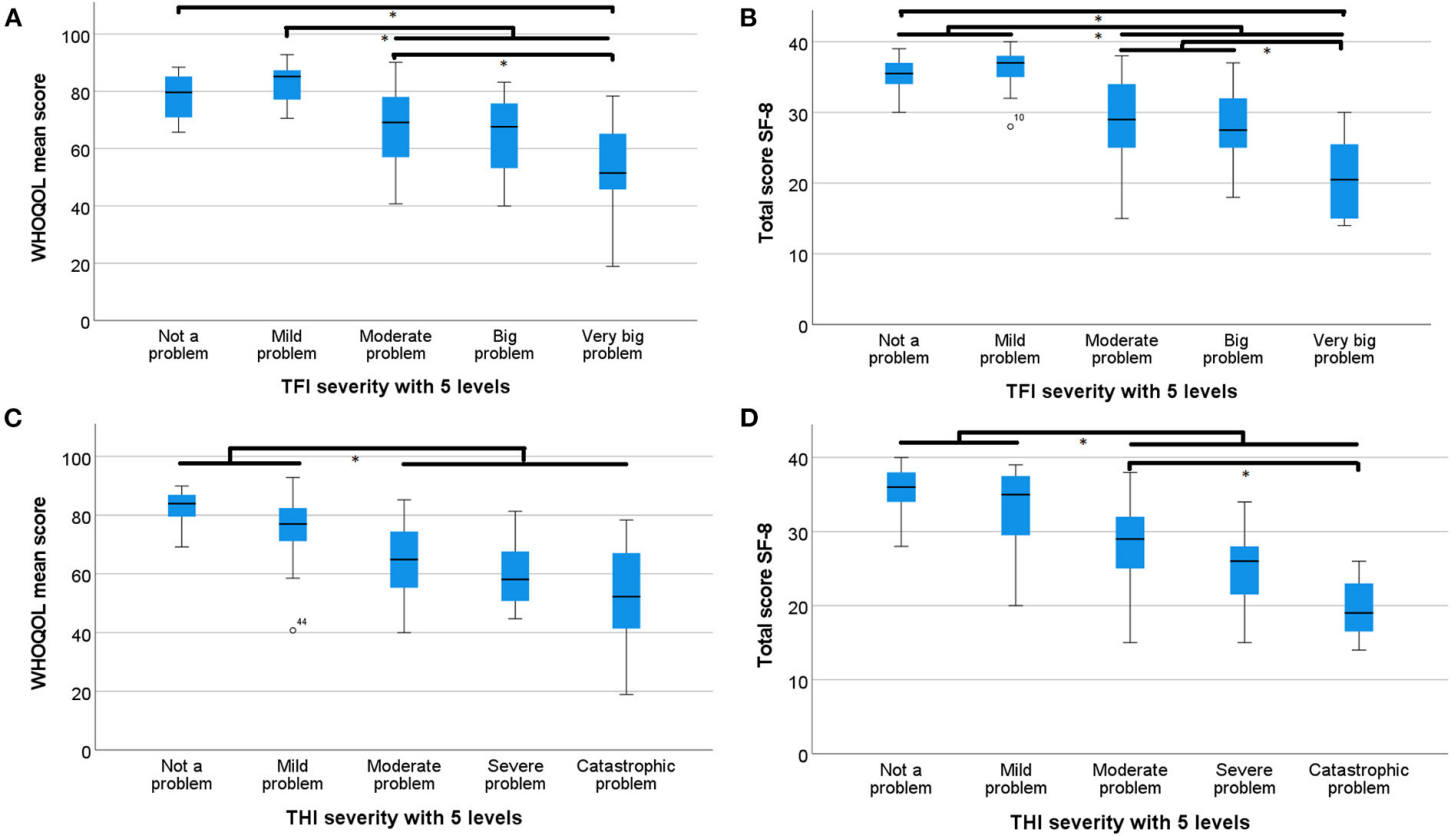
Boxplots of TFI and THI severity categories. TFI and THI severity categories and the median, minimum, maximum, and interquartile of WHOQOL-BREF and SF-8 are shown. * indicates a significant difference. **(A)** TFI severity categories vs WHOQOL-BREF scores, **(B)** TFI severity categories vs SF-8 scores, **(C)** THI severity categories vs WHOQOL-BREF scores, and **(D)** THI severity categories vs SF-8 scores.

To confirm the results, we calculated the difference in HRQoL between all grades of tinnitus severity, using the Tukey HSD test. The most interesting observations are mentioned in this paragraph. For all combinations of tinnitus and HRQoL questionnaires, no significant differences were observed between grades 1 and 2 (Figure 4). Notably, there was a significant difference between the first two grades (“low handicap” and “mild handicap”) and the three more severe grades using the THI (Figures 4C,D). Using the TFI, there was a significant decrease between grade 1 “not a problem” and grade 5 “very big problem” for both the WHOQOL-BREF mean and SF-8. No significant difference was noticed between the first grade and grades 2, 3, and 4 with the TFI (Figures 4A,B). On the other hand, when we looked at the difference between grade 2 “small problem” and the more severe grades (grades 3, 4, and 5), a significant result was observed (Figure 4A). In addition, the differences between grades 3 and 5 were also statistically significant for both the TFI compared to the WHOQOL-BREF (Figure 4A) and the THI compared to the SF-8 (Figure 4D). Similar to this finding, the difference between grade 3 and 4 was also statistically significant with grade 5 for the TFI with the SF-8 (Figure 4B).

## Discussion

In this study, we investigated the correlation between tinnitus questionnaires and HRQoL questionnaires in order to determine whether HRQoL questionnaires add value in assessing the HRQoL of tinnitus patients. Since it is often time-consuming to complete multiple questionnaires, the goal for this study was to shorten questionnaire completion to a minimum without loss of information. After calculating the four simple linear regressions with the tinnitus questionnaires as independent variables and the HRQoL questionnaires being the dependent outcomes, we observed four negative correlations, as expected. This finding confirms results from previous studies (26–28): the HRQoL decreases when the tinnitus severity increases. To find answers for our primary objective, we observed the strongest correlation between *THI* and *SF-8*. Our model suggested that the *THI* explains 54.4% of the HRQoL score of the *SF-8*, confirming the validity to use the *THI* in the assessment of the HRQoL of tinnitus patients (27). Similarly, a strong correlation between *TFI* and *SF-8* was demonstrated as well. Showing an explanation of 50.6 % of the variance in the HRQoL scores of the *SF-8*, the *TFI* is useful to evaluate the HRQoL of patients suffering from tinnitus. Here, we can conclude that the SF-8 covers approximately 50% of the same information that is already assessed in the tinnitus questionnaires. If we compare these values with those between the THI or TFI and WHOQOL (44.0, and 39.4%, respectively), these of the SF-8 turned out to be clearly higher. Therefore, using a combination of the SF 8 with a tinnitus questionnaire would not provide a lot of extra information regarding the HRQoL compared to the tinnitus questionnaires. The big advantage of this questionnaire is that it only includes eight questions, which is not time-consuming for both the patient and the physician. On the other hand, this also means that it does not provide specific information about the HRQoL of tinnitus patients. This latter was also confirmed when comparing the THI with the mental subdomain of both QoL questionnaires. If we look at the determination coefficients, we see that the mental subdomain of the SF-8 covers 62.9% of the THI, whereas the psychological subdomain of the WHOQOL-BREF only covers 39.5% of the same tinnitus questionnaire. Here, we can establish that the WHOQOL-BREF provides more insight in the psychological aspect of tinnitus compared to the SF-8.

Regarding the effect of the tinnitus questionnaires on the *WHOQOL-BREF*, the determination coefficient was decreased by approximately 10%. This implies that the *WHOQOL-BREF* gives more specific information about the HRQoL compared to the *SF-8*. The regression between the *TFI* and the *WHOQOL-BREF* mean was the weakest. This indicates that a combination of the *TFI* and the *WHOQOL-BREF* gives the most specific information about the HRQoL. In addition, the *WHOQOL-BREF* also has an added value compared to the *THI*. The *WHOQOL-BREF* contains more questions and more subdomains than the *SF-8*, which leads to a more thorough and more specific questionnaire in the assessment of the HRQoL. If more information about the impact of tinnitus, such as the social and environmental impact, is needed, we would recommend using the *WHOQOL-BREF* for the best understanding of the HRQoL.

To check what aspects of the HRQoL are evaluated in the tinnitus questionnaires, the regression coefficients of the *TFI* and *THI* with all subdomains of the HRQoL questionnaires were calculated. In both tinnitus questionnaires, the determination coefficient of the regression with the social relationship subdomain of the *WHOQOL-BREF* is very low. These regressions were also not significant. This confirms that there is no correlation between the “social relationship” subdomain and the tinnitus questionnaires, and that this subdomain is therefore insufficiently assessed in the *TFI* and *THI*. The regressions of the environment subdomain are significant but the determination coefficients are also low. As a result, the *THI* and *TFI* also concentrates less on this subdomain. As a previous study already established, we confirmed that the *THI* particularly focuses on the “physical health” and “psychological health”, and to a lesser extent on the “social relationship' and “environment” subdomain (27). This also is the case for the *TFI*, as investigated in this study. Furthermore, the correlation between the *SF-8* and the physical and psychological subdomains of the *WHOQOL-BREF* was very strong. This indicates that the *SF-8* HRQoL assessment questionnaire is particularly concerned with the physical and psychological effects of tinnitus, while the “social relationship” and “environment” subdomains are less pronounced.

Next, we wanted to investigate what aspects of HRQoL are included in the QoL subdomain of *TFI* and whether this subdomain gives sufficient information about the HRQoL. Here, we observed weak correlations between this subdomain and the subdomains social relationships and environment of the *WHOQOL-BREF*. This shows that the *TFI* QoL subdomain does not assess the social or environmental impact of tinnitus. Both the physical and psychological subdomain of the *WHOQOL-BREF* had a good correlation, but when both subdomains were combined, the correlation became even stronger. Moreover, the total score of the *SF-8*, which consists of a physical and mental component, also had a strong correlation. This implies that the QoL subdomain of the *TFI* predominantly focuses on the physical and psychological aspects of tinnitus and to a lesser extent on the social and environmental component. A previous study by Zeman et al. (27) showed that tinnitus strongly influences the physical and psychological subdomain of the HRQoL and to a lesser extent the social relationships and environment subdomain. This indicates that the QoL subdomain of the *TFI* gives sufficient information to assess the HRQoL in most cases.

In conclusion, if we want to assess the tinnitus burden on the HRQoL, focusing on the physical and psychological components, the QoL subdomain of the *TFI* provides sufficient information. Besides the fact that tinnitus primarily affects the physical health and causes psychological distress, the social aspect is also important for the assessment of HRQoL. By making it difficult to interact normally with other people, tinnitus can cause chronic distress that has an impact on a patient's daily activities (29). If we want to know more about the influence on the “social relationships” and “environmental” domain, it is recommended to use the *WHOQOL-BREF*, as the *TFI* QoL subdomain does not give enough information about these domains.

We also wanted to know what grade of tinnitus severity most affected the HRQoL. Examining the *TFI*, there was an interesting, significant decrease in the HRQoL scores from a “small problem” to a “moderate problem”. Similarly, the HRQoL in grades 3, 4, and 5 was significantly diminished compared to grades 1 and 2. Therefore, higher tinnitus severity scores are an indicator for clinicians to further evaluate the impact on a patient's HRQoL. This finding is in line with the studies of Zeman et al. (27) and Weidt et al. (30) where a strong correlation between the *THI* and *Beck Depression Inventory* (*BDI)* was observed. The study of Zeman et al. (27) demonstrated that high scores of the *THI* indicate the need to further evaluate the potential psychiatric comorbidities because severe and catastrophic tinnitus severity is related to depressive symptoms. Based on our results, it is recommended that a HRQoL questionnaire, preferably the *WHOQOL-BREF*, is used if a patient's tinnitus severity is in grade 3 ‘moderate problem’ or higher. If a patient's tinnitus severity is in grade 5 ‘very big problem’, it should be mandatory to evaluate the impact of tinnitus on the HRQoL by a HRQoL questionnaire. Since chronic tinnitus is associated with psychiatric disorders, it can result in depression and even suicide in extreme cases. Therefore, it is also recommended to further evaluate the degree of depression and anxiety disorders using the *Hospital Anxiety and Depression Scale* (*HADS*) or the *BDI*, when a patient has a tinnitus severity grade of 5 (31–33).

A possible point of concern of our study is that the mean score of the *WHOQOL-BREF* was used, because there is no global score of WHOQOL-*BREF* to assess overall HRQoL. Using the mean score assumes the weight of all subdomains is equal, but it might be the case that some subdomains interfere more with the HRQoL. Since there is no effective treatment for tinnitus, it is useful to compare the different interventions and to see what treatment options have the most benefit on the HRQoL. The *SF-8* has the advantage of being a short questionnaire, but it has no additional value for evaluating HRQoL when using the *TFI*. It would be interesting to investigate the additional benefit of the *Short-Form* 36 (*SF-36)*, consisting of 36 questions compared to eight questions, to see if this questionnaire gives more specific information about the impact on tinnitus related HRQoL. In addition, it might be helpful to investigate the relationship between the tinnitus questionnaires and the *Health Utility Index mark 3* (*HUI3)*. This questionnaire uses one total score to assess the HRQoL, which is more useful compared to the *WHOQOL-BREF* to compare scores between multiple HRQoL questionnaires. Moreover, it is useful in cost-utility analysis (CUA) as the questionnaire estimates quality-adjusted life-years (QALYs). Future research can investigate the added value of the *HUI* compared to the *WHOQOL-BREF*.

This study established that the *TFI* is a suitable questionnaire to assess both the tinnitus severity and HRQoL. It can be interesting to perform longitudinal studies in the future to check whether the *TFI* can detect changes in the HRQoL after treatment.

## Conclusion

We aimed at finding the best combination of questionnaires to recommend in clinical practice in order to reduce the time needed to complete multiple questionnaires without losing information. We established that the *SF-8* is limited to the physical and psychological aspects of the HRQoL, whereas the *WHOQOL-BREF* offers additional information about the impact on the environment and social relationships. In clinical practice, we would recommend using the *TFI* instead of the *THI*. Especially because the *TFI* has a separate subdomain that evaluates the HRQoL. This subdomain mainly assesses the physical and psychological domains of the HRQoL. Therefore, it gives a good overall view of the effect of tinnitus on the HRQoL. If it is important to know the social and environmental contribution to the HRQoL, it is recommended to use the *WHOQOL-BREF* questionnaire when assessing the HRQoL. Lastly, we analyzed at what grade of tinnitus severity the HRQoL is affected the most. When tinnitus becomes a moderate problem or worse, the *TFI* and *THI* are less useful to assess the HRQoL.

## Data availability statement

The raw data supporting the conclusions of this article will be made available by the authors, without undue reservation.

## Ethics statement

The studies involving human participants were reviewed and approved by BASEC-Nr. 2021-00361; Kanton Zürich; Kantonale Ethikkommission; Stampfenbachstrase 121; Postfach; 8090 Zürich. The patients/participants provided their written informed consent to participate in this study.

## Author contributions

VV had the idea for the project. NP, TK, and LV designed the project, arranged the ethical approval, and gathered data from patients. LV performed the study, analytic calculations, and interpretation of the results. TK, EC, and NP contributed to the interpretation of the results. The results were part of the master's dissertation of LV (Van Hoof, 2021, University of Antwerp). To make this data publicly available LV wrote this final manuscript with the support from NP, EC, and TK. NP, TK, and VV helped supervise the project. All authors provided critical feedback and helped shape the research, analysis and manuscript.

## Conflict of interest

The authors declare that the research was conducted in the absence of any commercial or financial relationships that could be construed as a potential conflict of interest.

## Publisher's note

All claims expressed in this article are solely those of the authors and do not necessarily represent those of their affiliated organizations, or those of the publisher, the editors and the reviewers. Any product that may be evaluated in this article, or claim that may be made by its manufacturer, is not guaranteed or endorsed by the publisher.
